# Synergistic Effect of Simvastatin and Romidepsin on Gamma-globin Gene Induction

**DOI:** 10.22074/cellj.2019.5589

**Published:** 2018-08-07

**Authors:** Hussain Habibi, Amir Atashi, Saeid Abroun, Mehrdad Noruzinia

**Affiliations:** 1 Department of Hematology, School of Medical Sciences, Tarbiat Modares University, Tehran, Iran; 2 Department of Medical Genetics, School of Medicine, Tarbiat Modares University, Tehran, Iran

**Keywords:** Beta-Thalassemia, Romidepsin, Sickle Cell Disease, Simvastatin

## Abstract

**Objective:**

Hemoglobinopathies such as beta-thalassemia and sickle cell disease (SCD) are inherited disorders that are caused by mutations in beta-globin chain. Gamma-globin gene reactivation can ameliorate clinical manifestations of beta- thalassemia and SCD. Drugs that induce fetal hemoglobin (HbF) can be promising tools for treatment of beta-thalassemia and SCD patients. Recently, it has been shown that Simvastatin (SIM) and Romidepsin (ROM) induce HbF. SIM is a *BCL11a* inhibitor and ROM is a HDAC inhibitor and both of these drugs are Food and Drug Administration (FDA)-approved for hypercholesterolemia and cutaneous T-cell lymphoma respectively. Our aim was to evaluate the synergistic effects of these drugs in inducing HbF.

**Materials and Methods:**

In our experimental study, we isolated CD34+ cells from five cord blood samples that were cultured in erythroid differentiation medium containing ROM and Simvastatin. Then Gamma-globin, *BCL11a* and HDAC gene expression were evaluated on the 7^th^and 14^th^day of erythroid differentiation by real-time polymerase chain reaction (PCR) and immunocytochemistry.

**Results:**

Our results showed that combination of SIM and ROM significantly increased Gamma-globin gene expression and inhibit *BCL11a* and HDAC expression compared to results of using each of them alone. SIM and ROM lead to 3.09- fold increase in HbF production compared to the control group. Also, SIM inhibited *BCL11a* expression (0.065-fold) and ROM inhibited HDAC1 expression (0.47-fold) as two important inhibitors of HbF production after birth.

**Conclusion:**

We propose combination therapy of these drugs may be ameliorate clinical manifestation in beta-thalassemia and SCD with at least side effects and reduce the need for blood transfusion.

## Introduction

Beta-thalassemia and sickle cell disease (SCD) are inherited disorders that are caused by mutations in beta-globin chain (1, 2), Patients suffering from these disorders need blood transfusion for survival; however, iron overload is an important side effect of frequent blood transfusion leading to liver diseases and heart attack (3, 4). For this reason, researchers have been looking for a better treatment since long ago. 

There are several approaches such as hematopoietic stem cell transplantation (HSCT), gene therapy and utilization of induced pluripotent stem cells (iPS), employed for treatment of beta-thalassemia and sickle cell anemia to ameliorate clinical symptoms of these conditions and reduce the need for blood transfusion. However, disadvantages of these approaches such as rare matched HLA donors, risk of graft versus of disease (GVHD) and virus vector transmission (5), Beta-thalassemia and SCD are the most frequent beta-hemoglobinopathies in the world, and a great number of countries that are affected by these diseases cannot perform HSCT and gene therapy easily (6, 7); so, researchers are looking for alternative therapies with lower risks and cost but higher chance of success. Typically, these patients have no symptoms at birth and clinical manifestations appear with HbF (α2γ2) switching to HbA (a2ß2) six months after birth (8-10). Scientists have found that high levels of HbF can ameliorate clinical symptoms in Beta-thalassemia and SCD patients (11). 

There are some ß-like thalassemia conditions such as Hereditary Persistence of Fetal Hemoglobin (HPFH), dßthalassemia and Corfu anemia that show elevated HbF and these patients do not have severe anemia and do not usually need blood transfusion (12-14). For three decades, scientists have focused to find pharmacological agents to reactivate Gamma-globin gene after birth (15-19). However, toxicity associated with these agents and other issues have restricted their use. Although hydroxyurea has been approved by the FDA as a HbF-inducing agent, its usage has been limited because it is not effective for all SCD patients and was effective only in few ß-thalassemia patients; also, it has a narrow therapeutic index due to decreased blood cells (especially neutrophils) count (20-23). Importantly, in countries with high prevalence of the mentioned hemoglobinopathies, utilization of pharmacological agents that can increase HbF in these patients is more affordable as compared to other methods. 

Studies have shown that there are several specific inhibitors for Gamma-globin expression after birth such as Histone deacetylase ½ (*HDAC1/2*) and B-cell lymphoma/leukemia 11a (*BCL11a*) (24-27). Macari et al. (28) showed that Simvastatin (SIM) as a *BCL11a* inhibitor can induce HbF in CD34+ obtained from peripheral blood cells. Also, Bates et al. (29) have noted that Romidepsin (ROM) can increase HbF in cutaneous T-cell lymphoma patients via inhibition of *HDAC1/2*. Many studies have introduced several agents such as BCL11a, HDAC1/2, KLF1, SOX2, MBD2, DRED, and DNMT that inhibit Gamma-globin expression after birth (25). Also, SIM and ROM are reported to be able to inhibit *BCL11a* and *HDAC1/2*, respectively, and were approved by the FDA for reduction of cholesterol, prevention of cardiovascular diseases (for Simvastatin) and treatment of cutaneous T-cell lymphoma (for Romidepsin). In this study, we evaluated the synergistic effect of ROM and SIM on induction of fetal hemoglobin in erythroid progenitors differentiated from cord blood stem cells. 

## Materials and Methods

### CD34+ cells separation and expansion

In this experimental study, umbilical cord blood samples (n=5) were collected at Sarem Hospital according to the guidelines of Medical Ethics Committee of Sarem Research Center and Tarbiat Modares University, Then, cord blood bags were transferred to the research laboratory of Sarem Hospital for further analysis. For isolation of mononuclear isolation cells, we used gradient separation (Ficoll-Paque plus GE Healthcare), and CD34+ cells were separated by a MACS procedure (Miltenyi Biotec, CD34 Micro Bead Kit, Germany). The mean number of cells in each bag was 1.2×10^6^. Separated cells were checked for CD34 expression by flowcytometry using FITC-conjugated anti-CD34. Also, the CD34+ cells were cultured in expansion medium [StemLine II serum-free culture medium, Sigma S0192 Containing stem cell factor (SCF) 100 ng/ml, IL3 1 ng/ml, thrombopoietin (TPO) 100 ng/ml, fms related tyrosine kinase 3 (Flt3) 100 ng/ml] for 4 days. We counted cells on the first and fourth day of expansion; number of cells of five bags on the first day was 6 million, which became 18 million after expansion. Also, we evaluated viability of cells on the 4th day by trypan blue staining. 

### MTT assay

We dissolved SIM (Cayman chemical company) and ROM (AOBIOUS) in dimethyl sulfoxide (DMSO) and then evaluated the cytotoxicity of SIM and ROM using MTT assay (Sigma, Germany). Here, 103 CD34+ cells were cultured and treated with different concentrations of SIM and ROM into each well in a 96-well microplate for 48 hours at 37oC with 95% humidity and 5% CO_2_. Then, the media was removed slowly (without removing cells) and 10 µl MTT reagent was added. After 4 hours of incubation at room temperature in the dark, 50 µl DMSO was added to solubilize the formazan particles. Then, optical density of each well was measured at 570 nm. Based on the MTT results, we used 10 µM/ ml SIM and 10 nM/ml Romidepsin, as they showed the greatest effectiveness with the least cytotoxicity at these concentrations.

### Erythroid differentiation, fetal hemoglobin induction and colony assay

Cells were cultured in erythroid differentiation medium [Stem line, Sigma S0192 containing erythropoietin (EPO) 3U/ml, SCF 100 ng/ml, IL3 5 ng/ ml, transferrin 5 ng/ml] for 7 and 14 days. Erythroid differentiation was confirmed by flowcytometry (Thermo Fisher, ABI, Attune^tm^ NxT Flow Cytometer) following incubation with PE-conjugated CD36 (Invitrogen, Denmark) and FITC-conjugated CD71 (Invitrogen, Denmark) monoclonal antibodies. According to the results, on the 14^th^ day, 78.5% and 63.3% of the differentiated cells expressed CD36 and CD71, respectively as erythroid lineage markers. We used five groups namely, control [that was culture in erythroid differentiation medium (EDM) only], ROM [that was treated with EDM+ROM (10 nM/ ml)], SIM [that was treated with EDM+SIM (10 µM/ ml)], Romidepsin/SIM (ROM/SIM, that was treated with EDM+ROM [10 nM/ml)+SIM (10 µM/ml)], and sodium butyrate [SB, that was treated with EDM+SB (100 µM/ml)]. The Changing of the condition medium for all the groups was performed once a week using 150 µl of fresh medium.

It should be noted that we used the SB group because, it was confirmed that SB can increase HbF, we used SB group to compare its results with the other groups. Colony assay evaluation was done using 1×10^3^ CD34+ cells that were vigorously mixed in 3 ml of methylcellulose medium (MethoCult H4230, Stem Cell Technologies) containing EPO 3 U/ml, SCF 100 ng/ml, IL3 5 ng/ml, transferrin 5 ng/ml. Then, the methylcellulose medium was placed into two 30-mm sterile petri dishes, each containing 1.5 ml of the medium, then, cells were spread slowly and subsequently placed in an incubator with 95% humidity and 5% CO_2_ at 37°C. After 14 days, the erythroid colonies were scored by a phase-contrast inversion microscope according standard colony assay protocol.

### RNA extraction and cDNA synthesis

RNA extraction was done by RNX-Plus solution for total RNA isolation (SinaClon Bioscience, Iran) and quality control procedure of the isolated RNA was undertaken with measurement of the absorbance at 260/280 nm by Biophotometer; the isolated RNA had an optical density between 1.9-2 with double distilled water used as blank. Afterward, cDNA was produced by a GeneAll kit (HyperScript^TM^ Reverse Transcriptase, South Korea); cDNA synthesis was done in 20 µl volume containing 3 µl extracted RNA, 1 µl dNTP, 1 µl oligo dT and 9 µl nuclease-free distilled water, which was heated to 65°C for 5 minutes and then placed on ice. After that, 6 µl of RT buffer including 10X RTase reaction buffer, DTT, HyperScriptTM Reverse Transcriptase and ZymAllTMRNase inhibitor were added and incubated for 50 minutes at 55°C followed by 5 minutes at 85°C. 

### Evaluation of gene expression using real-time polymerase chain reaction 

The primer sequences used to evaluate the expression levels of Gamma-globin, *BCL11a* and **HDAC1/2** genes, are mentioned in Table 1. The primers were designed using UCSC and NCBI databases and Gene runner software. At least one of the designed primers was pair spans an exon junction to avoid gene amplification on DNA. Also, cDNA synthesis by the GeneAll kit involved a step to assure that traces of contaminating DNA were removed. Then, polymerase chain reaction (PCR) was implemented in a 20-µl reaction in cap strip at 95°C for 10 minutes followed by 40 cycles at the denaturation temperature (30 seconds at 95°C), annealing temperature (30 seconds at 60°C) and extension temperature (30 seconds at 70°C). Each real-time PCR reaction was performed in duplicate. We used beta actin primers and control group to normalize our data by real time instrument (Applied biosystem, Step one, USA) and real time master mix (SYBR, Ampliqon real time master mix2x, high ROX). The ABI step one software was used to analyze data, including the cycle threshold (Ct), amplification plot and melting curve for each product. Moreover, efficiency of primers for each gene was evaluated by a standard curve generated using fourfold dilution series of synthesized cDNAs. Real-time results analysis was done by 2^-ΔΔct^ method and finally, statistical analysis for each gene was done by GraphPad Prism 7.

### Immunocytochemistry

Erythroid progenitors differentiated from cord blood on the 14^th^ day, were collected and washed with phosphate buffered saline (PBS) three times. Then, 105 cells were suspended in 1 ml of PBS, and cytospin cells were prepared on slide and fixed with absolute methanol (Merck, Germany) for 10 minutes and the slides were completely air-dried. Next, the fixed cells were permeabilized by 0.1% Triton X-100 at 18-25oC for 10 minutes. Then, we performed immunostaining by anti-HbF (BD Pharmingen™, Denmark) conjugated with fluorescein isothiocyanate (FITC) overnight at 4oC in the dark. Next, the stained cells were photographed using a fluorescence microscope (Motic BA410, with Moticam pro 282, Canada). We used newborn blood as positive sample for quality-control of staining protocol; also, we compared HbF induction between the control group (untreated) and the groups treated with ROM and Simvastatin.

## Results

### CD34+ Cells isolation and colony assay 

**Fig.1:**
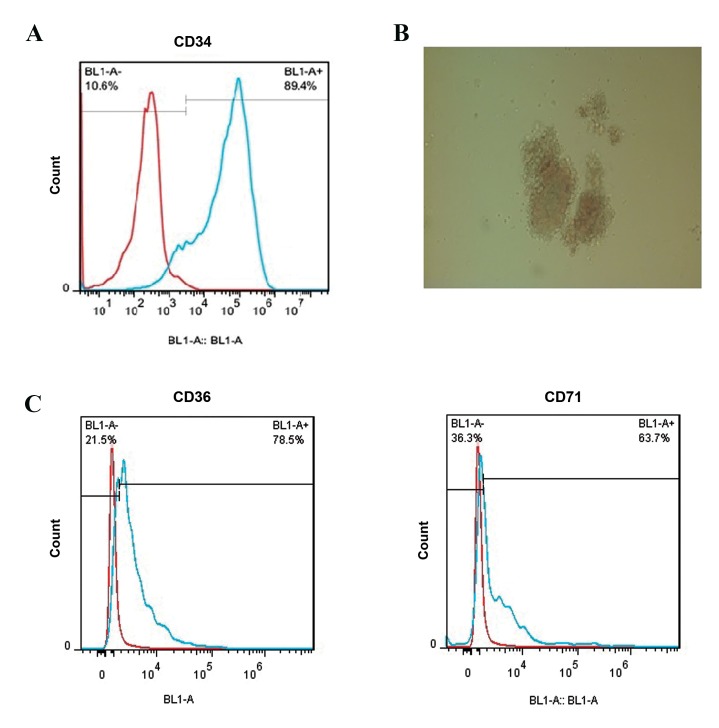
Flowcytometry result for CD34 isolation and erythroid differentiation. A. It was observed that 89.4% of the isolated cells of cord blood with MACS expressed CD34 as a HSC marker, B. The CD34+ isolated cells that were cultured in MethoCult medium, could differentiate into the erythroid commitment cells after 14 days (×100), and C. After 14 days 78.5 and 63.7% of CD34+ cells that were cultured in erythroid differentiation medium, expressed CD36 and CD71, respectively as erythroid markers. MACS; Magnetic-activated cell sorting and HSC; Hematopoietic stem cell.

The CD34+ cells were isolated by MACS positive selection and evaluation of CD34+ cells purity was done with anti CD34-FITC and flowcytometry. Flowcytometry analysis showed that 89.4% of the cells isolated from cord blood expressed CD34 as a HSC marker (Fig .1A). The viability of isolated cells was 99% as assessed by trypan blue staining (only 1% of cells were stained by trypan blue and the rest of them were alive). Also, the result of colony assay on the 14^th^ day confirmed that the isolated cells can differentiate into erythroid commitment cell (Fig .1B). The flowcytometry analysis on the 14^th^ day showed that the hematopoietic stem cells (HSCs) that were cultured in the erythroid differentiation medium expressed CD36 (78.5%) and CD71 (63.7%) as erythroid markers; thus, HSCs isolated from cord blood could differentiate into erythroid progenitors cells (Fig .1C). 

**Table 1:** Real time polymerase chain reaction primer sequences


Primers	Primer sequencing (5′-3′)	Size (bp)

Gamma-globin	F: TGTGGAAGATGCTGGAGGAGA	71
	R: CAAAGAACCTCTGGGTCCATG	
*BCL11a*	F: CGCAGCGACACTTGTTCTTC	84
	R: GCTTCCATCCGAAAACTGCC	
*HDAC1*	F: CAGCCTAGTGCGGTGGTC	108
	R: GACAAATTCCACACACTTGGC	
*HDAC2*	F: CCTAGTGCTGTGGTATTACAGTG	100
	R: CTTCTACACATTTAGCATGACCT	
*Beta Actin*	F: GGAGAAGAGCTACGAGCTGCC	117
	R: TGGATGCCACAGGACTCCAT	


### Relative Gamma-globin gene expression

**Fig.2:**
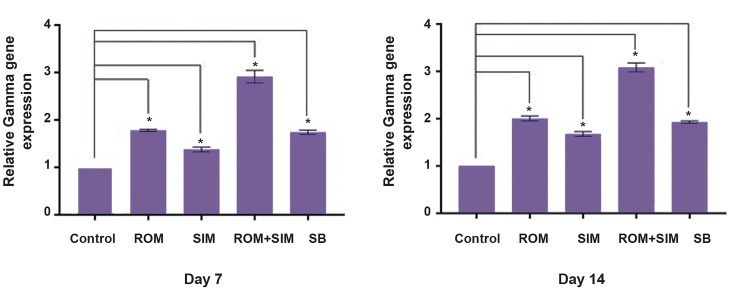
SIM and ROM can increase Gamma-globin gene expression, but the combination of these drugs synergistically induced Gamma-globin gene expression. Also, ROM/SIM can induce Gamma-globin to higher levels compared to SB, as a confirmed HbF inducer. *; P<0.05, ROM; Romidepsin, SIM; Simvastatin, SB; Sodium butyrate, and HbF; Fetal hemoglobin.

Evaluation of Gamma-globin gene expression by real time PCR showed that SIM and ROM treatment led to 1.7-fold increase in Gamma-globin gene expression compared to untreated group, on the 7^th^ and 14^th^ day. However, when we used SIM and ROM together (SIM 10 µM/ml and ROM 10 nM/ml), 3-fold increment in gamma gene mRNA was observed compared to untreated group, on the 7^th^ and 14^th^ day (Fig .2). These findings indicated that SIM and ROM can increase Gamma-globin gene expression synergistically (P<0.05). Also, no significant differences were observed in gamma expression between the 7^th^ and 14^th^ days; thus, we could have finished our study on the 7^th^ day. However, to compare the results obtained on the 7^th^ day with those of the 14^th^ day, we continued the experiment until the 14^th^ day. In this study, we used SB as a drug which was shown to induce Gamma-globin gene induction, and compared its results with those of ROM and SIM treatment. Our results showed that ROM is more marked upregulation of Gamma-globin gene expression compared to SB.

### Relative *BCL11a* gene expression

**Fig.3:**
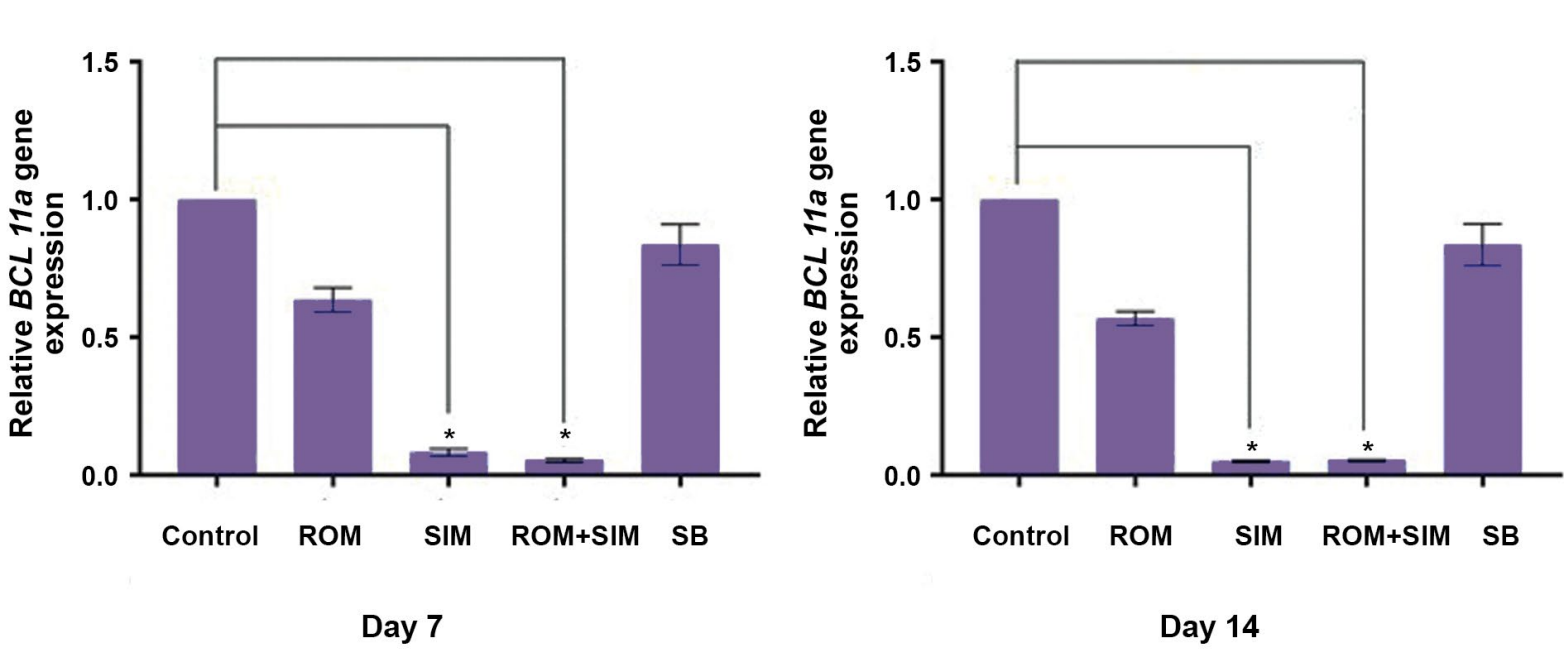
Results of the 7^th^ and 14^th^ day showed that only SIM and ROM/SIM can significantly inhibit *BCL11a* gene transcription, as compared to the control group, while ROM and SB did not show significant inhibition of *BCL11a*. *; P<0.05, ROM; Romidepsin, SIM; Simvastatin, and SB; Sodium butyrate.

Evaluation of *BCL11a* gene expression by real time step one software showed that ROM no significantly inhibited *BCL11a* (mean: 0.6-fold higher than the control group), whereas SIM treatment led to a significant inhibition of *BCL11a* mRNA transcription (mean: 0.065fold higher than that of the control group, P<0.05). Also, consistent with our study, Macari et al. (28) reported that SIM can inhibit *BCL11a* gene expression. In addition, our results showed that the combination of ROM and SIM significantly downregulated *BCL11a* compared to untreated group (P<0.05, Fig .3).

### Romidepsin and Simvastatin effects on HDAC1 and *HDAC2* expression 

**Fig.4:**
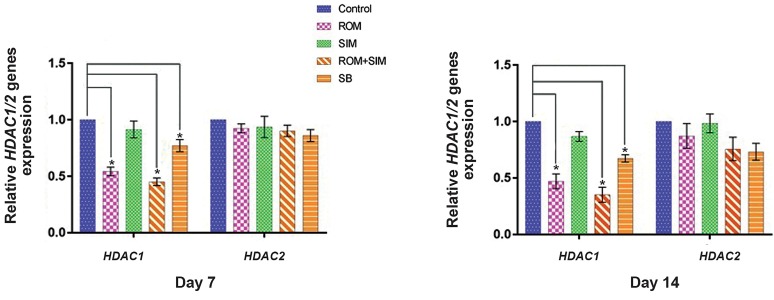
Effect of ROM, SIM and ROM/SIM on CD34+ cells showed that ROM and ROM/SIM can only downregulate HADC1, but not *HDAC2*. Seemingly, Gamma-globin upregulation is related to HDAC1 and *BCL11a* downregulation not *HDAC2*. *; P<0.05, ROM; Romidepsin, SIM; Simvastatin, SB; Sodium butyrate.

Relative quantitative real time PCR was done for the CD34+ cells that had been treated with ROM, SIM and ROM/SIM using the primers mentioned in Table 1 for *HDAC1/2*. Our results showed that HDAC1 expression was significantly downregulated by ROM and ROM/SIM (P<0.05), but not by SIM alone. In addition, results of the quantitative real time PCR showed that neither ROM, SIM nor ROM/SIM had significant effects on the expression of *HDAC2*. It seems that the effect of the mentioned drugs on Gamma-globin gene expression only was mediated by their effects on HDAC1 (Fig .4) and *BCL11a* inhibition (Fig .3). 

### ROM, SIM and ROM/SIM increased HbF in the treated cells

**Fig.5:**
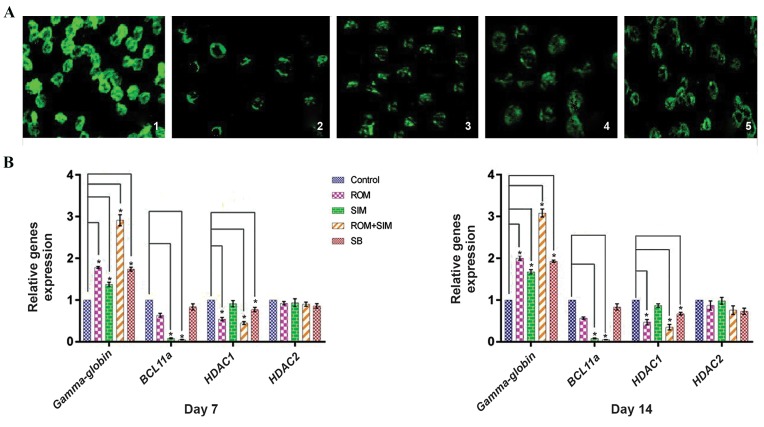
Immunofluorescence staining for HbF production and Real time evaluation for *BCL11a, HDAC* and Gamma-globin genes expression. A. Results of immunofluorescence staining showed that ROM and SIM can induce HbF production in erythroid progenitors differentiated form cord hematopoietic stem cells: 1) Newborn blood was used as positive control for immunofluorescence staining, 2) Untreated group, 3) ROM group, 4) SIM group, 5) ROM/SIM group (×100) and B. CD34+ cells were treated with ROM, SIM and ROM/SIM for 7 and 14 days. Results of gene expression showed that ROM and SIM can induce Gamma-globin gene expression and downregulate *BCL11a* and HDAC1 genes expression on the 7^th^ and 14^th^ day, but the combination of ROM and SIM can induce Gamma-globin gene transcription synergistically by downregulation of *BCL11a* and *HDAC1* genes on the 7^th^ and 14^th^ day. We also found that ROM and SIM had no effect on *HDAC2* gene expression. *; P<0.05, HbF; Fetal hemoglobin, ROM; Romidepsin, SIM; Simvastatin, and SB; Sodium butyrate.

HbF was evaluated using FITC-conjugated anti-F and fluorescence microscopy. In our study, we used two controls as follows: i. Newborn blood was used as positive control of fluorescence staining and ii. Untreated group was used to compare its results in terms of HbF production with those of the treated groups (ROM, SIM, and ROM/SIM). Results of fluorescence staining showed that both ROM and SIM can increase HbF in erythroid progenitors differentiated form cord hematopoietic stem cells. These findings are consistent with results of Makala et al. (30) for ROM and Macari et al. (28) for SIM. However, the results revealed greater HbF production, when ROM/SIM (ROM 10 nM/ml and SIM 10 µM/ ml) were added to erythroid differentiation medium, as compared to results obtained from using ROM and SIM alone (Fig .5A). These results suggest that ROM and SIM increase HbF production synergistically; however, results of gene expression by real time analysis in our study are more evident. Real time results showed that ROM/SIM significantly downregulated *BCL11a* and HDAC1 while caused upregulation of HbF expression (2.91-fold higher than the untreated group) on the 7^th^ and (3.09-fold higher than the untreated group) 14^th^ days (Fig .5B).

## Discussion

ß-thal and SCD are the most frequent hemoglobinopathies in the world. Almost more than 80% of patients with ß-thal and SCD are born in non-industrial and developing countries, while these countries do not have adequate facilities for prenatal screening, diagnosis, treatment and proper management of these patients (31, 32). 

Traditional therapies such as frequent blood transfusion can lead to iron overload, liver disease, heart attack, risk of virus transmission and alloimmunization (33). Scientists offer alternative therapeutic approaches such as HSCT, gene therapy and iPS usage, to ameliorate clinical symptoms and reduce need for blood transfusion. But, these approaches are hardly available in non-industrial countries. Thus, in the last three decades, researchers have tried to present therapeutic approaches with lower risk and cost and easily available (15, 18, 34-36).

HbF inducing drugs are the best approach to ameliorate clinical symptoms of ß-thal and SCD. Atashi et al. (18) confirmed that SCF and tumor growth factor-beta (TGF-ß) can induce HbF in CD133+ cells. Also, these transcription factors have synergistic effects for HbF induction; however, SCF and TGF-ß are not approved by the FDA and there is major concern that their long-term usage may be carcinogenic, in addition to the fact that SCF and TGF-ß usage are not cost effective. Ahmadvand et al. (37) showed that thalidomide at 100 µM concentration, can induce HbF in CD133+ cells differentiated into erythroid lineage 1.5- fold higher than the control group. Our results showed that SIM (10 µM) and ROM (10 nm) can induce HbF production 3.09-fold higher than the control group. Kukreja et al. (38) introduced some natural agents that can induce HbF. However, there is still need for novel agents that safely induce HbF and decrease need for blood transfusion. Constantoulakis et al. (39) showed SB and 5-azacytidine have synergistic effect for HbF induction; However, there are concerns over the carcinogenic potential of 5-azacytidine. Also, SB and its derivatives have disadvantages such as requirement of high doses (15-20 g/day), short half-life, and unpleasant smell (40).

Currently, hydroxyurea is the only drug approved by the FDA for these patients though it possesses side effects such as undesirable long-term carcinogenesis and blood suppression and being not effective for all patients. For these reasons, efforts are continuing to introduce better drugs. Since inhibition of HbF occurs in multiple pathways after birth, we decided to evaluate the effect of the combination of two FDA-approved drugs with different mechanism for HbF induction (10). Our results showed that ROM as a HDAC inhibitor and SIM as a *BCL11a* inhibitor can considerably induce HbF in hematopoietic stem cells. The combination of SIM and ROM caused simultaneous downregulation of *BCL11a* and HDAC1 but significantly increased HbF expression. Similarly, Elizabeth et al. showed that combination of SIM and t-butylhydroquinone increases Gamma-globin expression 3.2-fold higher than the control group. Currently, SIM is using reduction of cholesterol and prevention of cardiovascular diseases. Also, no serious side effect has been reported following long-period usage of Simvastatin, yet. Moreover, ROM is using for cutaneous T-cell lymphoma treatment and both drugs are approved by the FDA (29). In addition, our results showed that the combination of these drugs increases HbF. Thus, we suggest their concurrent use for HbF induction as a therapeutic approach in ß-thal and SCD patients.

## Conclusion

HbF inducing is the best approach for treatment of patients with ß-thal and SCD, and hydroxyurea is the only FDA-approved drug for HbF induction, but it cannot be used for all of ß-thal patients and it has side effects such as suppression of blood counts. Results of our study showed that the combination of ROM and SIM simultaneously caused downregulation of HDAC1 and *BCL11a* while induced Gamma-globin gene expression. These drugs are FDA-approved and thus, can be used together to ameliorate clinical symptoms in ß-thal and SCD patients. We hope ROM and SIM combination therapy may lead to promising results in ß-thal and SCD patients with least side effects and reduce need for blood transfusion.
